# Japan's development assistance for health: Historical trends and prospects for a new era

**DOI:** 10.1016/j.lanwpc.2022.100403

**Published:** 2022-02-22

**Authors:** Shuhei Nomura, Lisa Yamasaki, Kazuki Shimizu, Cyrus Ghaznavi, Haruka Sakamoto

**Affiliations:** a Department of Health Policy and Management, School of Medicine, Keio University, 35 Shinanomachi, Shinjuku-ku, Tokyo 160-8582, Japan; b Department of Global Health Policy, Graduate School of Medicine, The University of Tokyo, Tokyo, Japan; c Tokyo Foundation for Policy Research, Tokyo, Japan; d Better Co-Being, Tokyo, Japan; e Global Health Governance Study Group, Japan Center for International Exchange, Tokyo, Japan; f School of Medicine, Nagasaki University, Nagasaki, Japan; g Medical Education Program, Washington University School of Medicine in St Louis, Saint Louis, United States

**Keywords:** Japan, Development assistance for health, Health system strengthening, Non-communicable diseases, Disease burden, National income, COVID-19

## Abstract

The year 2020 marked an important turning point in Japan's global health policy. While the global health community has been suffering serious damage to sustainable health financing due to the COVID-19 pandemic, an independent commission on Japan's Strategy on Development Assistance for Health (DAH) launched an ambitious policy recommendation to double the amount of Japan's DAH during the post-COVID-19 era. This paper examines historical trends in DAH in Japan over the past 30 years based on published literature and comprehensive DAH tracking data and highlights priority areas for discussion on how DAH can be advanced to ensure equitable and efficient use of limited resources to support the achievement of the Sustainable Development Goals, including universal health coverage and pandemic preparedness, in low- and middle-income countries. Priority areas for discussion include: how and where to focus DAH for equitable health gains; how to provide DAH to support health system strengthening, including pandemic preparedness; and clarifying the role of DAH in global health functions.

## Introduction

The coronavirus disease 2019 (COVID-19) pandemic has sparked an unprecedented level of interest in the past, present, and future of global health financing, in part because of the enormous and ongoing costs to countries around the world, regardless of socioeconomic level, of responding to the pandemic.^1^ Global gross domestic product in 2020 fell globally by 3.1% from the previous year.^2^ It is projected to grow 5.9% in 2021,^2^ but with disproportionate vaccination coverage, the accelerating spread of SARS-CoV-2 variants, and the exhaustion of public health resources, the projection is subject to a great deal of uncertainty.^3^ The financial impact of the health crisis has led to long-term economic stagnation in some countries, and progress toward achieving the Sustainable Development Goals (SDGs), including universal health coverage (UHC), has stalled or reversed.^4^, ^5^, ^6^

Development assistance for health (DAH) is a key component of foreign policy for many donor countries and has an important role in supporting improvements in population health and helping to build human capital in low- and middle-income countries (LMICs).^7^^,^^8^ DAH then can enhance economic development, which in turn promotes and supports health security and the development and access to global public goods.^9^ In addition, in response to the health and economic crises caused by COVID-19, DAH has played a unique role in financing emergency health systems in many countries and brought about the rapid expansion of necessary health services.^10^

In 2000, Japan hosted the G8 Kyushu-Okinawa Summit, led the establishment of the Global Fund to Fight AIDS, Tuberculosis and Malaria (Global Fund), and highlighted infectious disease control as a major global health agenda. It became a monumental opportunity as G8 countries confirmed the need for additional funding and international partnerships. Two decades later, the year 2020 marked a major turning point in Japan's DAH strategy. To date, Japan has made significant contributions to the global health debate in the areas of infectious diseases, health system strengthening, UHC, and health emergency response.^11^^,^^12^ The Basic Design for Peace and Health (BDPH), which was formulated in 2015 as an issue-specific policy for the health sector in the Development Cooperation Charter (a fundamental policy document that details the development cooperation policies of Japan), will soon be revised for the post-COVID-19 era. The current BDPH outlines three basic policies: establishing resilient global health governance that can respond to public health emergencies and disasters; promoting seamless utilization of essential health and medical services and UHC throughout lifecycle; and leveraging Japanese expertise, experience, and medical products and technology.^13^

In order to contribute to this revision, an independent commission chaired by Yasuhisa Shiozaki, former Minister of Health, Labor and Welfare of Japan, with members from the National Diet, government, private sector, academia, and civil society, was set up to review Japan's global health strategy and propose recommendations.^14^ Doubling the amount of Japan's DAH within five years was set as the new global health contribution target, as one of several proposals, including clarification of the governmental command post for DAH strategy development and implementation, and active participation in the governance of international development agencies. However, there remains no clear direction as to how DAH can be advanced moving forward.

This paper has two objectives. First, we assess the historical trends of Japan's DAH over the last 30 years. Then, we highlight priority areas for discussion that donor countries, including Japan, should address to keep the future of DAH on track to support the achievement of the SDGs (including UHC) as well as pandemic preparedness in LMICs (DAH recipient countries). Priority areas for discussion include how and where to focus DAH for equitable health gains, how to provide DAH to support health system strengthening, including pandemic preparedness, and clarifying DAH's role in funding the core functions of global health, such as global public goods provision and the management of cross-border externalities. They are developed in the context of the demographic and epidemiological transitions in LMICs, the new challenges revealed by the COVID-19 pandemic, and a global political shift toward increasing prioritization of donor funding into aid for global functions. We believe that this paper will inform the revision of BDPH by proposing new venues of DAH allocation for Japan to explore.

## Methods

In addition to the published literature, we used data on the latest estimates of DAH for each recipient country published by the Institute for Health Metrics and Evaluation (IHME).^15^^,^^16^ It relies primarily on open databases on disbursement at project levels (including the Organisation for Economic Cooperation and Development's Creditor Reporting System (OECD CRS)), and, where possible, on direct data collection for individual agencies. For the purposes of this study, DAH, as defined by IHME, refers to disbursements (the amount actually distributed), rather than pledges or commitments (the amount the donors agreed to make available), made by donors to maintain and improve health in LMICs. These data include which health focus areas were targeted, which countries received DAH, and even through which international development agencies and bilateral agencies the funds were distributed. Health focus areas (including health system strengthening as well as pandemic preparedness) relevant to the projects were uniquely defined by IHME through keyword searches of project descriptions downloaded from the databases. The most recent estimates are for 1990–2018, and those for 2019 and 2020 are projections. DAH is reported in inflation-adjusted 2020 USD. Unless otherwise indicated, this study presents the DAH for 2020 excluding funds for COVID-19 as COVID-19-related disbursements constitute an emergency response, unlike the DAH strategy for peacetime. Also, unless otherwise noted, health system strengthening refers to sector-wide approaches and does not include efforts for a specific health focus area (i.e. diagonal approach). By IHME's definition, pandemic preparedness is a subset of health systems strengthening. Therefore, our working definition of pandemic preparedness is much narrower than the extensive discussion of pandemic preparedness often found in the literature.

Data on gross national income (GNI) and population size were extracted from the World Bank's World Development Indicators.^17^^,^^18^ Data on disability-adjusted life years (DALYs) – an overall health loss indicator that takes into account premature death and disability – were extracted from the Global Burden of Disease Study 2019 (GBD 2019).^19^^,^^20^

### Scale and scope of Japan's DAH in the past and present

**Figure 1:**
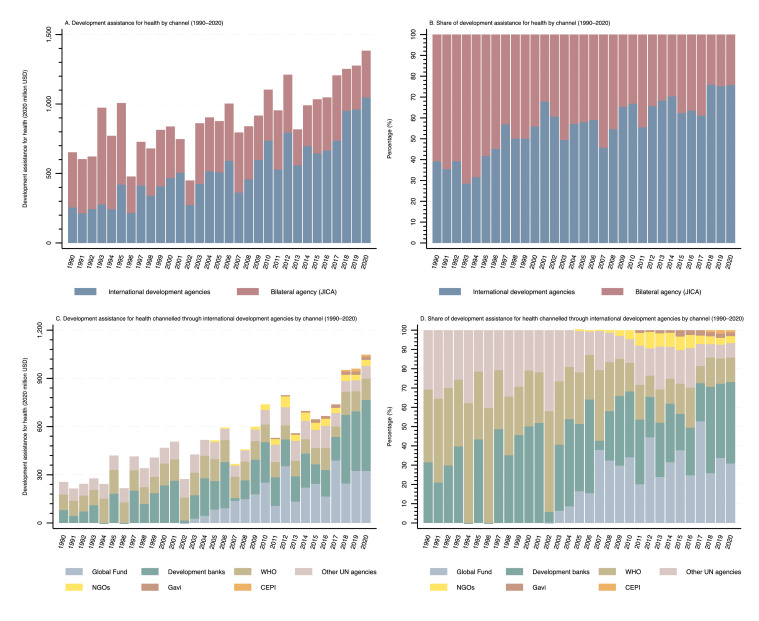
**Development assistance for health by channel, 1990–2020**. CEPI=Coalition for Epidemic Preparedness Innovations. Gavi=Gavi, the Vaccine Alliance. Global Fund=Global Fund to Fight AIDS, Tuberculosis and Malaria. JICA=Japan International Cooperation Agency. NGO=Non-governmental Organisation. WHO=World Health Organization. Development banks includes International Development Association, Inter-American Development Bank, Asian Development Bank, and African Development Bank. Other United Nations (UN) agencies include the United Nations Children's Fund (UNICEF), the United Nations Population Fund (UNFPA), Unitaid, and the Joint United Nations Programme on HIV/AIDS (UNAIDS).

DAH from Japan has maintained an increasing trend since 1990, the beginning of the analysis period (Figure 1A), with an average annual growth rate of 6.2% during this period, growing at a scale of 19.2 million USD annually. In 2018, 1.3 billion USD of DAH was provided. 20 years ago, the share of DAH through international development agencies was roughly 50%, whereas in the last few years it has increased from 60% to over 70% (Figure 1B). These increases are mostly explained by the increase in DAH channeled through international development agencies (especially Global Fund) (Figure 1C); the Global Fund now accounts for about 30% of DAH channeled through international development agencies. Other channels with particularly high DAH shares are multilateral development banks (such as the International Development Association and the Asian Development Bank), World Health Organization (WHO), and other UN agencies (Figure 1C). The share of DAH channeled through these international development agencies since 2000 has averaged 32.0%, 21.8%, and 17.5%, respectively (Figure 1D).

The share of DAH through the Coalition for Epidemic Preparedness Innovations (CEPI) and Gavi, the Vaccine Alliance (Gavi) is not very large (about 1.1% for CEPI and 2.0% for Gavi in 2018). CEPI is a public-private partnership founded in 2016 that has played a vital role in research and development (R&D) as well as the manufacturing of vaccines through global collaboration, in which Japan was involved as one of the founding members; Gavi is a public-private partnership to promote procurement and delivery of vaccines to LMICs.

**Figure 2:**
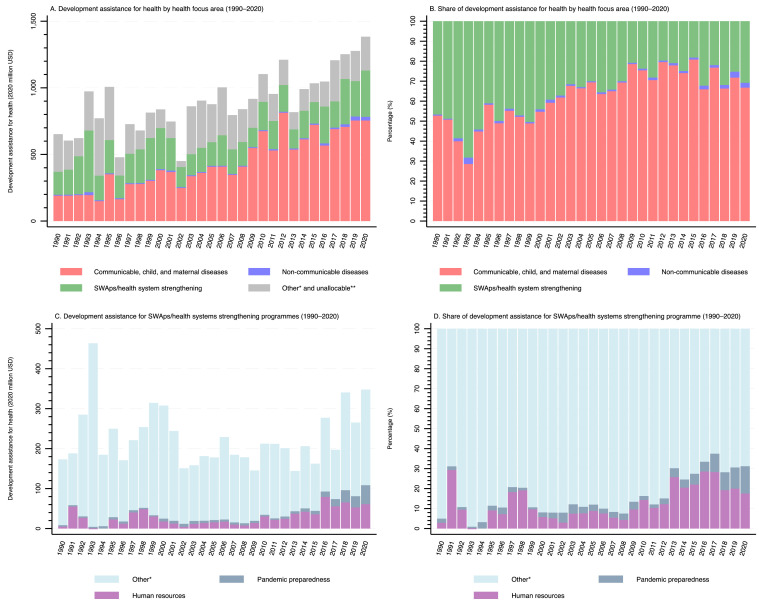
**Development assistance for health by health focus area, 1990–2020.** SWAps=sector-wide approaches. *”Other” captures development assistance for health for which source information was available but was not identified as being allocated to any of the health focus areas listed. Health assistance for which no health focus area information was available was designated as unallocable.

By health focus area, the increase in DAH since 2000 has mostly corresponded to communicable, child, and maternal diseases control (Figure 2A). This may be due to the fact that the Global Fund is an organization whose main mission is combatting the big three infectious diseases: HIV/AIDS, tuberculosis, and malaria. Excluding the ‘other’ and ‘unallocable’ health focus areas, it now has a share of about 70%, while it is noteworthy that only about 2% of DAH are allocated for non-communicable diseases (NCDs) (Figure 2B). Among health system strengthening, which have recently had a share of about 30%, pandemic preparedness has accounted for only about 5%, except in the last few years (projections) (Fig 2C and D). Note that pandemic preparedness, as defined by IHME, is a subset of health system strengthening and refers to epidemiological surveillance, contact tracing and control, biosafety measures, early warning, etc.^15^

The channels with a particularly large share of DAH from Japan in health system strengthening are Japan International Cooperation Agency (JICA) (43.9% in 2018), WHO (39.6%), development banks (33.3%), and NGOs (19.4%). Among health system strengthening, WHO (32.8%) and JICA (9.0%) have the largest share in pandemic preparedness (as a subset of health system strengthening).

**Figure 3:**
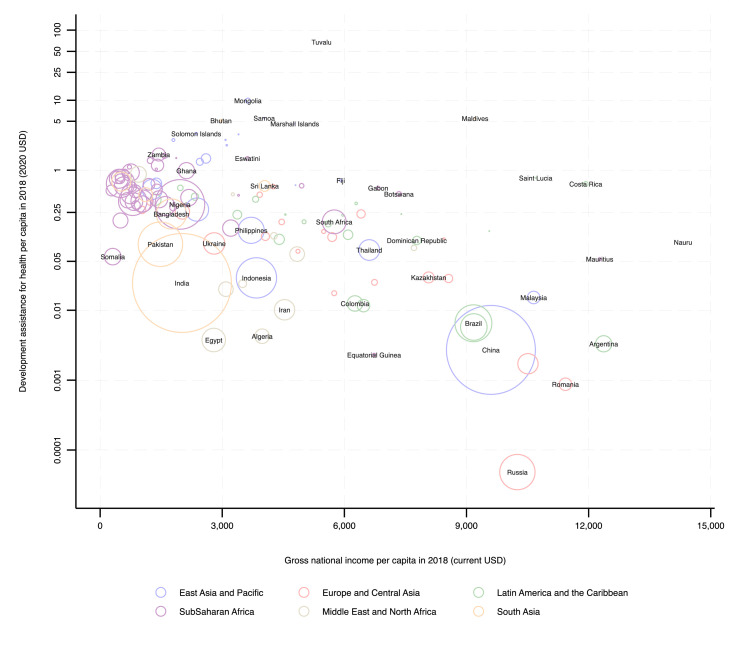
**Development assistance for health received per person by gross national income per person, 2018.** Each dot represents a recipient country, and dot sizes correspond to national disease burdens, as measured by DALYs in 2018. DALYs=disability-adjusted life-years.

**Figure 4:**
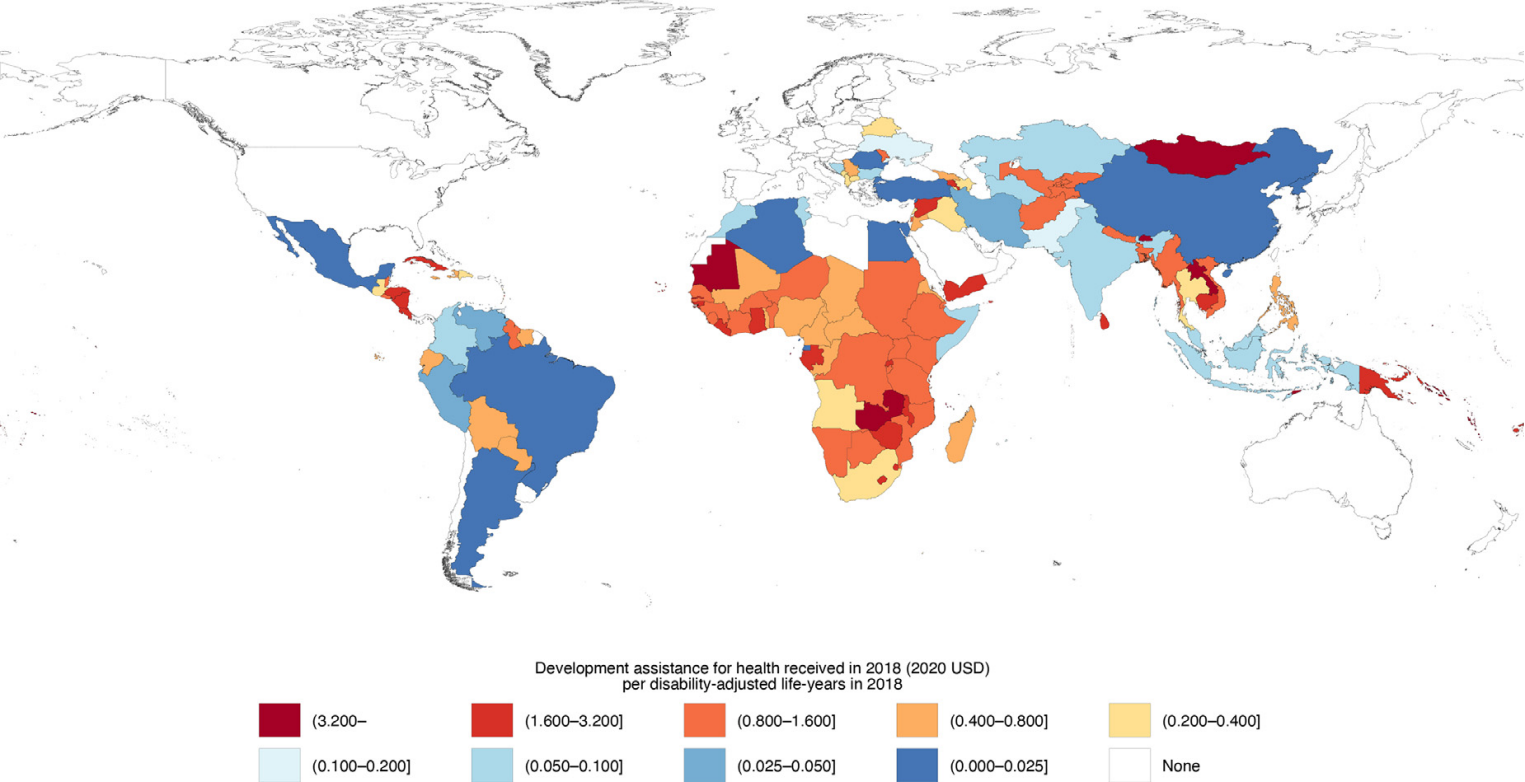
**Development assistance for health received per disability-adjusted life-years, 2018**. Development assistance for health, in inflation-adjusted 2020 USD, per disability-adjusted life-year lost in 2018. Countries in white did not receive any development assistance for health in 2018.

While donors, including Japan, have their own methodologies for deciding how to distribute DAH, the criteria for assessing equity in the distribution of their DAH can include national income (an indicator of economic needs) and disease burden (an indicator of health needs).^21^^,^^22^ Figure 3 shows that the relationship between Japan's DAH per capita and GNI per capita in 2018 is weak. Although the general tendency is for higher GNI per capita to be associated with lower DAH per capita, there is wide variation in the amount of DAH received even among countries with similar levels of GNI. DAH per DALYs in 2018 also varied widely across recipient countries (Figure 4).

### Priority areas for discussion

The SDG era is characterized by the expansion of global goals to include more interdependent and multisectoral areas of focus. In particular, climate change, natural disasters, which are increasing in scale and frequency, as well as refugee crises and conflicts have the potential to directly and indirectly affect health needs, and the DAH landscape must change accordingly.^23^ It is not just a matter of increasing the amount of DAH, but a paradigm shift is needed to ensure that limited resources are provided in a more equitable, efficient, and sustainable manner, and that global health goals are achieved in partnership with recipient countries. Specifically, we have established three priority areas for discussion on how DAH should be delivered.

### How to focus DAH for equitable health gains

There are many possible reasons for the variation in the allocation of DAH from donor countries, including Japan, that cannot be explained by economic or health needs. Donors are likely to focus on the expected impact of DAH in terms of changes in health or determinants of health (including health service coverage), the ability (beyond financial factors) of countries to implement and expand services, and the fair distribution of resources and services.^21^ In addition, DAH allocations from donor countries may be guided by a number of additional factors, such as historical diplomatic relations, geographic proximity, strategic political interests, and especially in the case of bilateral aid, trade-related considerations.^24^^,^^25^

A fundamental challenge for donors, including Japan, is critically reviewing each country's own criteria and rationale for DAH allocation.^26^ For Japan, in order to meet the SDGs and the UHC principle of leaving no one behind, addressing inequality is essential, and this requires moving beyond eligibility based solely on national income to criteria that include national disease burden, socioeconomic status, national inequality, and each country's ability to address them. It is also important to consider how to deliver aid to marginalized populations in each country, including migrants and refugees. The size of most vulnerable populations should also be considered in DAH allocation calculations and strategies.^26^

### Where to focus DAH for equitable health gains

There is considerable debate about which health areas DAH from donors should prioritize. DAH allocations from major donors, including Japan, are known not to be closely linked to disease burden.^26^ While communicable, child, and maternal diseases continue to be the main focus of Japan's DAH, NCDs accounted for 33.9%, 55.2%, and 78.9% of the total burden of disease (measured in DALYs) in low, lower-middle, and upper-middle income countries (defined by World Bank) in 2019.^19^ There are important arguments for why Japan and other major donor countries should increase their investments in NCDs.^27^^,^^28^ For example, it was highlighted by the GBD 2019 study that some NCDs, which have continued to increase globally over the past three decades, are risk factors for severe COVID-19 illness and are driving the increase in deaths from COVID-19.^29^ This suggests that insufficient action has been taken to address key risk factors for NCDs such as obesity, hypertension, high blood sugar, smoking, and alcohol, and that the world needs to take concerted action.^30^^,^^31^ This might include supporting the development and implementation of new policies to promote population health, such as regulation, taxation, and subsidies.^32^^,^^33^

The disease burden of most NCDs increases with increasing age, such as cardiovascular diseases, neoplasms, diabetes, neurological disorders (e.g. Alzheimer's disease), mental disorders, etc.^19^ Even in LMICs, more attention needs to be paid to the needs of aging populations in order to effectively respond to long-term changes in disease structure.^34^ Importantly, greater investment in NCDs does not justify sacrificing funding for communicable, child, and maternal diseases. Evaluating where to direct DAH from donors should reflect an assessment of the potential health benefits and cost-effectiveness of aid programmes,^35^, ^36^, ^37^ the comparative advantages of the aid implementation agencies or channels, and considerations tailored to the context of each recipient country's health needs.

### How to provide DAH to support health system strengthening, including pandemic preparedness

While more diseases and sectors are becoming targets for DAH investments from donors, without meaningful investment in strengthening key health system pillars (e.g. WHO's building blocks of service delivery, health workforce, health information systems, information systems, access to essential medicines, financing, and leadership/governance^38^), health gains are less likely to be sustained and achieving UHC and other SDGs will be more costly.^39^^,^^40^ Aligning DAH spending with recipient countries’ priorities will also support their efforts to improve population health outcomes and achieve global health goals. Investing in health system strengthening and broader system support is one way to do this and can help align long-term goals.^26^^,^^41^ Donors must consider that there are mismatches between current DAH allocations and recipient countries’ priorities (e.g. based on national health strategic plans).^42^^,^^43^

COVID-19 highlights an important role that health systems play in ensuring health security.^40^ Reducing mortality from COVID-19 will require, at the very least, a strong health system with sufficient capacity to test, trace, and treat patients and the ability to obtain and deliver the vaccine quickly and efficiently.^44^ The impact of COVID-19 on providing essential health services has been significant, largely because of the need to re-organize health care resources, including health care workers, equipment at health care facilities, services, data, and funding, to meet the urgent demands of the pandemic, making it much more difficult for patients to safely access health care services.^45^

### What is the role of DAH in global health functions

We need to think hard about the role of DAH from donors, including Japan, in funding what Schäferhoff et al. (2015) called the ‘core functions’ of global health, including the provision of global public goods and the management of cross-border externalities.^46^ These functions include R&D of new therapeutics and vaccines, development of a global system for prevention, early detection and containment of large-scale epidemics, efforts to combat antimicrobial resistance, response to unhealthy food products, and measures to mitigate the impact of climate change on health and health care delivery systems. According to Schäferhoff et al. (2019), globally, only about 16.1% of DAH (including R&D) was invested in global public goods in 2013, which increased to 17.1% in 2015 and then declined to 15.4% in 2017.^47^ In addition, in 2013, 5.5% of DAH was invested in the management of cross-border externalities, including pandemic preparedness and control of antimicrobial resistance, which increased to 10.2% in 2015 and then declined to 7.2% in 2017.^47^ Fully addressing the impact of a pandemic in most LMICs will require not only a strong health system that can respond, but also affordable access to critical tools such as vaccines. As highlighted by the G20 High Level Independent Panel on Financing the Global Commons for Pandemic Preparedness and Response June 2021 report,^48^ concerted action to invest in global public goods is essential to mitigate the health and economic losses associated with the COVID-19 pandemic and to prevent and rapidly respond to the next global health emergency. In addition to CEPI, which plays an important role in generating global public goods for those in need, including vaccine development, relatively large DAH shares from donors in global health functions are provided by WHO (62% in 2013), the Joint United Nations Programme on HIV/AIDS (UNAIDS) (40%), the United Nations Population Fund (UNFPA) (22%), and Gavi (20%).^46^ As of 2018, Japan had a very small share of its DAH channeled through both CEPI and Gavi. Additionally, at present, there is no established methodology for monitoring funding for global public goods, and it is necessary to establish such a mechanism in the future, including the agreed definition of global public goods and appropriate data collection.

## Conclusion

According to the previous literature, Japan has disbursed a total of 2.3 billion USD toward addressing the health-related effects of COVID-19 in LMICs.^15^ This figure is the largest among all donor countries and international development agencies and is equivalent to 1.8 times Japan's DAH in peacetime (2018), proving that Japan can rapidly scale up its resources as needed. However, it cannot be ruled out that the economic downturn associated with the COVID-19 pandemic may affect policy decisions by donor countries seeking to maintain record development assistance resources for LMICs.^49^ The United Kingdom (UK), for example, has already cut its aid budget in order to prioritize addressing domestic challenges,^50^ raising concerns that this will affect the health systems of the countries that have been receiving UK aid, leaving them vulnerable to disruption of foreign aid.^51^^,^^52^

Nevertheless, as the COVID-19 pandemic has progressed over time, there have been even more calls for assistance to LMICs.^53^ These requests are not limited to COVID-19, but also in response to efforts to achieve global goals such as the SDGs including UHC and to address ever-changing global health challenges such as climate change, refugee crises, conflict, terrorism, and emerging infectious diseases.^23^ Therefore, priority areas for discussion to ensure equitable and efficient use of limited resources should include: how and where to focus DAH for equitable health gains; how to provide DAH to support health system strengthening, including pandemic preparedness; and clarifying the role of DAH in global health functions, including global public goods provision.

## Declaration of interests

SN reports grants from the Bill & Melinda Gates Foundation and the Ministry of Education, Culture, Sports, Science and Technology of Japan (21H03203), during the conduct of the study. The findings, interpretations, and conclusions expressed in the paper are entirely those of the authors.
